# Supplementary material for: Drug repurposing for aging research using model organisms
Source: Aging Cell. 2017 Jun 16;16(5):1006–15. doi: 10.1111/acel.12626 (PMC5595691; doi:10.1111/acel.12626)
Supplement: Supplementary file 7 — Data S1 Zip‐Archive of all report cards. [file ACEL-16-1006-s007.zip › RC_COA.pdf]

# COA

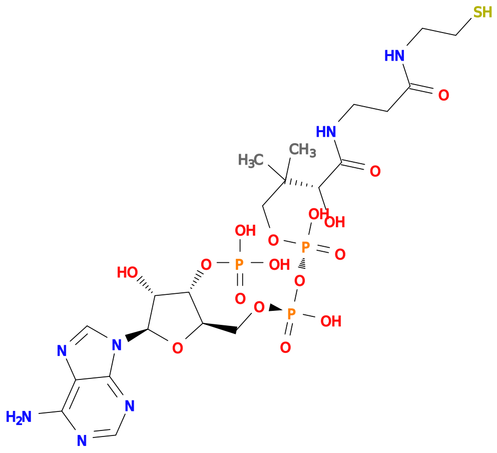


**Database identifiers**

| | |
|---|---|
| ChEMBLCompound | CHEMBL1213327 |
| CHEBI | 15346 |

## Ranking

| | Rank | Score |
|---|---|---|
| Drosophila | 399/697 | 0.363 |
| C. elegans | 575/591 | 0.0 |

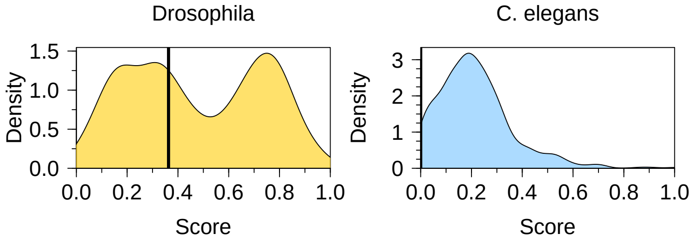


| | Ageing implication | Domain conservation | Binding site conservation | Binding affinity | Bioavailability | Lipinski | Promiscuity | Purchasability | Drug approval | **Total** |
|---|---|---|---|---|---|---|---|---|---|---|
| Drosophila | 0.81 | 0.928 | 1.0 | 0.764 | (0.9) | -0.15 | -0.004 | 0.0 | 0.0 | 0.363 |
| C. elegans | 0.81 | 0.926 | 1.0 | 0.764 | 0.201 | -0.15 | -0.004 | 0.0 | 0.0 | 0.0 |

## Names

- **coenzyme A**
- 3'-phosphoadenosine-(5')diphospho(4')pantatheine
- CoA
- CoASH
- HSCoA

## Roles

coenzyme

## Status

| | |
|---|---|
| Approved drug (according to ChEMBL) | No |
| Number of Rule of 5 violations | 3 |
| Binding affinity to original target in log units (RF-Score prediction) | 6.17 |
| Burns *C. elegans* bioavailability prediction | -11.0 |

## Compound Target Characteristics

### 3-hydroxy-3-methylglutaryl-coenzyme A reductase

Best gene implication in ageing for this target family came from gene P51639 annotated in UniProt release 2014_02. Annotation GO 7568 (aging) was Inferred from Expression Pattern

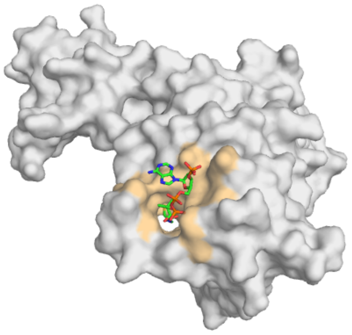

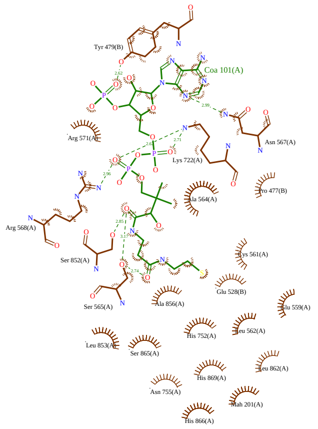

| protein | amino acids contacts (binding site) | | | | | | | | | | | | | | | | | | | | | |
|---|---|---|---|---|---|---|---|---|---|---|---|---|---|---|---|---|---|---|---|---|---|---|
| PDB:1dq8:chainA:P04035 | P | Y | E | E | G | C | L | A | S | N | R | R | V | K | H | N | S | L | A | L | - | - | - |
| PDB:1dqa:chainA:P04035 | P | Y | E | E | G | C | L | A | S | N | R | R | V | K | H | N | S | L | A | L | S | H | H |
| sp:P04035:HMDH_HUMAN | P | Y | E | E | G | C | L | A | S | N | R | R | V | K | H | N | S | L | A | L | S | H | H |
| sp:P51639:HMDH_RAT | P | Y | E | E | G | C | L | A | S | N | R | R | V | K | H | N | S | L | A | L | S | H | H |
| sp:Q01237:HMDH_MOUSE | P | Y | E | E | G | C | L | A | S | N | R | R | V | K | H | N | S | L | A | L | S | H | H |
| tr:E1JIU8:E1JIU8_DROME | P | H | E | E | G | A | L | A | S | N | R | K | V | K | H | N | S | L | A | L | S | H | H |
| tr:A9UN11:A9UN11_DROME | P | H | E | E | G | A | L | A | S | N | R | K | V | K | H | N | S | L | A | L | S | H | H |
| sp:P14773:HMDH_DROME | P | H | E | E | G | A | L | A | S | N | R | K | V | K | H | N | S | L | A | L | S | H | H |
| tr:Q19207:Q19207_CAEEL | K | R | E | E | G | A | L | A | S | N | R | N | T | R | H | N | S | L | A | L | S | H | L |
| sp:P12684:HMDH2_YEAST | P | Y | E | E | G | C | L | A | S | M | R | K | V | K | H | N | S | L | A | L | S | H | H |
| sp:P12683:HMDH1_YEAST | P | Y | E | E | G | C | L | A | S | M | R | K | V | K | H | N | S | L | A | L | S | H | H |

| protein | whole protein ident | whole protein simil | domain-based ident | domain-based simil | contact-based ident | contact-based simil |
|---|---|---|---|---|---|---|
| PDB:1dq8:chainA:P04035 | 1.0 | 1.0 | 1.0 | 1.0 | 0.87 | 0.73 |
| PDB:1dqa:chainA:P04035 | 1.0 | 1.0 | 1.0 | 1.0 | 1.0 | 1.0 |
| sp:P04035:HMDH_HUMAN | 1.0 | 1.0 | 1.0 | 1.0 | 1.0 | 1.0 |
| sp:P51639:HMDH_RAT | 0.93 | 0.98 | 0.94 | 0.99 | 1.0 | 1.0 |
| sp:Q01237:HMDH_MOUSE | 0.93 | 0.98 | 0.95 | 0.99 | 1.0 | 1.0 |
| tr:E1JIU8:E1JIU8_DROME | 0.44 | 0.74 | 0.56 | 0.82 | 0.87 | 0.87 |
| tr:A9UN11:A9UN11_DROME | 0.44 | 0.74 | 0.56 | 0.82 | 0.87 | 0.87 |
| sp:P14773:HMDH_DROME | 0.44 | 0.74 | 0.56 | 0.82 | 0.87 | 0.87 |
| tr:Q19207:Q19207_CAEEL | 0.24 | 0.43 | 0.45 | 0.73 | 0.7 | 0.72 |
| sp:P12684:HMDH2_YEAST | 0.3 | 0.63 | 0.57 | 0.83 | 0.91 | 0.93 |
| sp:P12683:HMDH1_YEAST | 0.29 | 0.61 | 0.56 | 0.8 | 0.91 | 0.93 |

### Hmgcr (FBgn0263782) associated phenotypes

cold sensitive, developmental rate defective, germline clone, heat sensitive, lethal - all die before end of second instar larval stage, partially, partially lethal - majority die, rescuable maternal effect, segment polarity phenotype, small body, somatic clone, some die during pupal stage, some die during

second instar larval stage, some die during third instar larval stage

(Information from FlyBase)

**Hmgcr (UniProt:E1JIU8) annotation**
**Pathway**: Metabolic intermediate biosynthesis; (R)-mevalonate biosynthesis; (R)-mevalonate from acetyl-CoA: step 3/3. (RuleBase:RU361219).
**Subcellular location**: Endoplasmic reticulum membrane (RuleBase:RU361219); Multi-pass membrane protein (RuleBase:RU361219).

(Information from UniProt)

**Hmgcr (UniProt:A9UN11) annotation**
**Pathway**: Metabolic intermediate biosynthesis; (R)-mevalonate biosynthesis; (R)-mevalonate from acetyl-CoA: step 3/3. (RuleBase:RU361219).
**Subcellular location**: Endoplasmic reticulum membrane (RuleBase:RU361219); Multi-pass membrane protein (RuleBase:RU361219).

(Information from UniProt)

**Hmgcr (UniProt:P14773) annotation**
**Function**: Synthesis of mevalonate for the production of non-sterol isoprenoids, which are essential for growth differentiation. Provides spatial information during embryogenesis to guide migrating primordial germ cells (the pole cells) from the ectoderm to the mesoderm. Also required for association of the pole cells with the gonadal mesoderm. (PubMed:9853754).
**Enzyme regulation**: The activity of HMG-CoA-reductase is suppressed by exogenous mevalonate. (PubMed:3136321).
**Pathway**: Metabolic intermediate biosynthesis; (R)-mevalonate biosynthesis; (R)-mevalonate from acetyl-CoA: step 3/3.
**Subcellular location**: Endoplasmic reticulum membrane; Multi-pass membrane protein.
**Tissue specificity**: Highly expressed in embryonic gonadal mesoderm, where expression is initially broad, and then becomes restricted to a segmental pattern at stage 11. Expression is then further restricted to a cluster of cells in each of parasegments 10, 11 and 12, corresponding to the developing gonadal mesoderm. Not expressed in pole cells. (PubMed:9853754).

(Information from UniProt)

**hmgr-1 (WBGene00017268) associated phenotypes**
lethal, sterile

(Information from WormBase)

**hmgr-1 (UniProt:Q19207) annotation**
**Pathway**: Metabolic intermediate biosynthesis; (R)-mevalonate biosynthesis; (R)-mevalonate from acetyl-CoA: step 3/3. (RuleBase:RU361219).
**Subcellular location**: Endoplasmic reticulum membrane (RuleBase:RU361219); Multi-pass membrane protein (RuleBase:RU361219).

(Information from UniProt)

**Histone acetyltransferase ESA1**

Best gene implication in ageing for this target family came from gene Q92794 annotated in UniProt release 2014_02. Annotation GO subterm of 7568 (aging) was Inferred from Mutant Phenotype

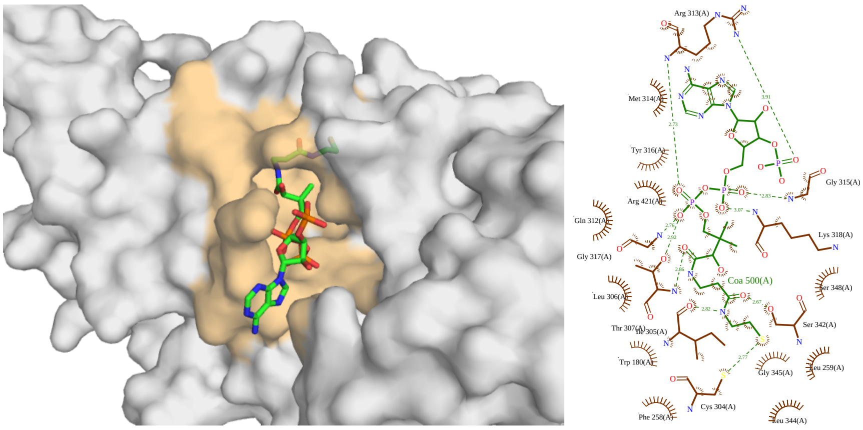


| protein | amino acids contacts (binding site) | | | | | | | | | | | | | | | | | | |
|---|---|---|---|---|---|---|---|---|---|---|---|---|---|---|---|---|---|---|---|
| PDB:1fy7:chainA:Q08649 | W | F | L | C | I | L | T | Q | R | M | G | Y | G | K | L | S | L | G | S | R |
| PDB:1mj9:chainA:Q08649 | W | F | L | S | I | L | T | Q | R | M | G | Y | G | K | L | S | L | G | S | R |
| PDB:1mja:chainA:Q08649 | W | F | L | X | I | L | T | Q | R | M | G | Y | G | K | L | S | L | G | S | R |
| PDB:3to7:chainA:Q08649 | W | F | L | C | I | L | T | Q | R | M | G | Y | G | K | L | S | L | G | S | R |
| PDB:3to9:chainA:Q08649 | W | F | L | C | I | L | T | Q | R | M | G | Y | G | K | L | S | L | G | S | R |
| tr:C9K0F9:C9K0F9_HUMAN | W | F | L | C | I | M | I | Q | R | K | G | Y | G | R | L | S | L | G | S | P |
| tr:A5PLL3:A5PLL3_HUMAN | W | F | L | C | I | M | I | Q | R | K | G | Y | G | R | L | S | L | G | S | P |
| tr:A5PKX7:A5PKX7_HUMAN | W | F | L | C | I | M | I | Q | R | K | G | Y | G | R | L | S | L | G | S | P |
| sp:Q92794:KAT6A_HUMAN | W | F | L | C | I | M | I | Q | R | K | G | Y | G | R | L | S | L | G | S | P |
| tr:Q3V1G6:Q3V1G6_MOUSE | W | F | L | C | I | M | I | Q | R | K | G | Y | G | R | L | S | L | G | S | P |
| tr:Q3UPM9:Q3UPM9_MOUSE | W | F | L | C | I | M | I | Q | R | K | G | Y | G | R | L | S | L | G | S | P |
| tr:G3X940:G3X940_MOUSE | W | F | L | C | I | M | I | Q | R | K | G | Y | G | R | L | S | L | G | S | P |
| tr:D0IQI5:D0IQI5_DROME | W | F | L | C | I | L | T | Q | R | Q | G | Y | G | R | L | S | L | G | S | R |
| tr:Q9W1A9:Q9W1A9_DROME | W | F | L | C | I | L | T | Q | R | Q | G | Y | G | R | L | S | L | G | S | R |
| tr:L8E6K3:L8E6K3_CAEEL | W | F | L | C | I | M | T | Q | E | M | G | L | G | R | L | S | L | G | A | K |
| tr:Q21789:Q21789_CAEEL | W | F | L | C | I | M | T | Q | E | M | G | L | G | R | L | S | L | G | A | K |
| tr:D7SFM3:D7SFM3_CAEEL | W | F | L | C | I | M | T | Q | E | M | G | L | G | R | L | S | L | G | A | K |
| tr:K8ESF3:K8ESF3_CAEEL | W | F | L | C | I | M | T | Q | E | M | G | L | G | R | L | S | L | G | A | K |
| tr:G5EGU3:G5EGU3_CAEEL | W | F | L | C | I | M | T | Q | E | M | G | L | G | R | L | S | L | G | A | K |
| tr:K8ES81:K8ES81_CAEEL | W | F | L | C | I | M | T | Q | E | M | G | L | G | R | L | S | L | G | A | K |
| tr:G5EGP0:G5EGP0_CAEEL | W | F | L | C | I | M | T | Q | E | M | G | L | G | R | L | S | L | G | A | K |
| tr:K8F7U3:K8F7U3_CAEEL | W | F | L | C | I | M | T | Q | E | M | G | L | G | R | L | S | L | G | A | K |
| tr:G5EBH5:G5EBH5_CAEEL | W | F | L | C | I | M | T | Q | E | M | G | L | G | R | L | S | L | G | A | K |
| tr:K8ERR8:K8ERR8_CAEEL | W | F | L | C | I | M | T | Q | E | M | G | L | G | R | L | S | L | G | A | K |
| sp:Q08649:ESA1_YEAST | W | F | L | C | I | L | T | Q | R | M | G | Y | G | K | L | S | L | G | S | R |

| protein | whole protein ident | simil | domain-based ident | simil | contact-based ident | simil |
|---|---|---|---|---|---|---|
| PDB:1fy7:chainA:Q08649 | 1.0 | 1.0 | 1.0 | 1.0 | 1.0 | 1.0 |
| PDB:1mj9:chainA:Q08649 | 1.0 | 1.0 | 1.0 | 1.0 | 0.95 | 0.95 |
| PDB:1mja:chainA:Q08649 | 1.0 | 1.0 | 1.0 | 1.0 | 0.95 | 0.92 |
| PDB:3to7:chainA:Q08649 | 1.0 | 1.0 | 1.0 | 1.0 | 1.0 | 1.0 |
| PDB:3to9:chainA:Q08649 | 0.99 | 1.0 | 0.99 | 1.0 | 1.0 | 1.0 |
| tr:C9K0F9:C9K0F9_HUMAN | 0.16 | 0.29 | 0.46 | 0.76 | 0.75 | 0.85 |
| tr:A5PLL3:A5PLL3_HUMAN | 0.19 | 0.4 | 0.46 | 0.76 | 0.75 | 0.85 |
| tr:A5PKX7:A5PKX7_HUMAN | 0.14 | 0.28 | 0.46 | 0.76 | 0.75 | 0.85 |
| sp:Q92794:KAT6A_HUMAN | 0.08 | 0.16 | 0.46 | 0.76 | 0.75 | 0.85 |
| tr:Q3V1G6:Q3V1G6_MOUSE | 0.13 | 0.28 | 0.46 | 0.76 | 0.75 | 0.85 |
| tr:Q3UPM9:Q3UPM9_MOUSE | 0.12 | 0.25 | 0.46 | 0.76 | 0.75 | 0.85 |
| tr:G3X940:G3X940_MOUSE | 0.08 | 0.16 | 0.46 | 0.76 | 0.75 | 0.85 |
| tr:D0IQI5:D0IQI5_DROME | 0.13 | 0.3 | 0.4 | 0.71 | 0.9 | 0.94 |
| tr:Q9W1A9:Q9W1A9_DROME | 0.06 | 0.14 | 0.4 | 0.71 | 0.9 | 0.94 |
| tr:L8E6K3:L8E6K3_CAEEL | 0.21 | 0.47 | 0.36 | 0.69 | 0.7 | 0.88 |
| tr:Q21789:Q21789_CAEEL | 0.2 | 0.46 | 0.35 | 0.68 | 0.7 | 0.88 |
| tr:D7SFM3:D7SFM3_CAEEL | 0.09 | 0.22 | 0.35 | 0.68 | 0.7 | 0.88 |
| tr:K8ESF3:K8ESF3_CAEEL | 0.09 | 0.22 | 0.35 | 0.68 | 0.7 | 0.88 |
| tr:G5EGU3:G5EGU3_CAEEL | 0.1 | 0.22 | 0.35 | 0.68 | 0.7 | 0.88 |
| tr:K8ES81:K8ES81_CAEEL | 0.09 | 0.22 | 0.35 | 0.68 | 0.7 | 0.88 |
| tr:G5EGP0:G5EGP0_CAEEL | 0.08 | 0.2 | 0.35 | 0.68 | 0.7 | 0.88 |
| tr:K8F7U3:K8F7U3_CAEEL | 0.08 | 0.2 | 0.35 | 0.68 | 0.7 | 0.88 |
| tr:G5EBH5:G5EBH5_CAEEL | 0.08 | 0.2 | 0.35 | 0.68 | 0.7 | 0.88 |
| tr:K8ERR8:K8ERR8_CAEEL | 0.08 | 0.19 | 0.35 | 0.68 | 0.7 | 0.88 |
| sp:Q08649:ESA1_YEAST | 1.0 | 1.0 | 1.0 | 1.0 | 1.0 | 1.0 |

**enok-RA (UniProt:D0IQI5) annotation**
**Subcellular location**: Nucleus ECO:0000256—RuleBase:RU361211.

(Information from UniProt)

**enok (FBgn0034975) associated phenotypes**
female semi-sterile, germline clone, neuroanatomy defective, size defective, somatic clone

(Information from FlyBase)

**enok (UniProt:Q9W1A9) annotation**
**Subcellular location**: Nucleus ECO:0000256—RuleBase:RU361211.

(Information from UniProt)

**lsy-12 (WBGene00045419) associated phenotypes**
egg laying defective, locomotion variant, loss of asymmetry ASE, protruding vulva, reduced brood size, sick

(Information from WormBase)

**lsy-12 (UniProt:L8E6K3) annotation**
**Subcellular location**: Nucleus ECO:0000256—RuleBase:RU361211.

(Information from UniProt)

**lsy-12 (UniProt:Q21789) annotation**
**Subcellular location**: Nucleus ECO:0000256—RuleBase:RU361211.

(Information from UniProt)

**lsy-12 (UniProt:D7SFM3) annotation**
**Subcellular location**: Nucleus ECO:0000256—RuleBase:RU361211.

(Information from UniProt)

**lsy-12 (UniProt:K8ESF3) annotation**
**Subcellular location**: Nucleus ECO:0000256—RuleBase:RU361211.

(Information from UniProt)

**lsy-12 (UniProt:G5EGU3) annotation**
**Subcellular location**: Nucleus ECO:0000256—RuleBase:RU361211.

(Information from UniProt)

**lsy-12 (UniProt:K8ES81) annotation**
**Subcellular location**: Nucleus ECO:0000256—RuleBase:RU361211.

(Information from UniProt)

**lsy-12 (UniProt:G5EGP0) annotation**
**Subcellular location**: Nucleus ECO:0000256—RuleBase:RU361211.

(Information from UniProt)

**lsy-12 (UniProt:K8F7U3) annotation**
**Subcellular location**: Nucleus ECO:0000256—RuleBase:RU361211.

(Information from UniProt)

**lsy-12 (UniProt:G5EBH5) annotation**
**Subcellular location**: Nucleus ECO:0000256—RuleBase:RU361211.

(Information from UniProt)

**lsy-12 (UniProt:K8ERR8) annotation**
**Subcellular location**: Nucleus ECO:0000256—RuleBase:RU361211.

(Information from UniProt)

**Histone acetyltransferase p300**

Best gene implication in ageing for this target family came from gene P34545 via mapping the annotation from WormBase WBGene00000366 annotated in WormBase 2014-03-11. Annotation GO 8340 (determination of adult lifespan) was Inferred from Mutant Phenotype

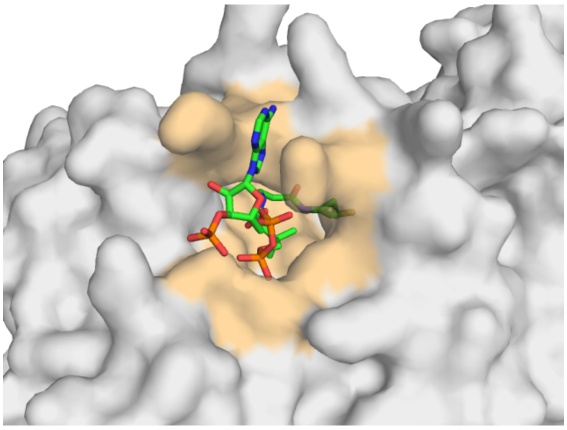

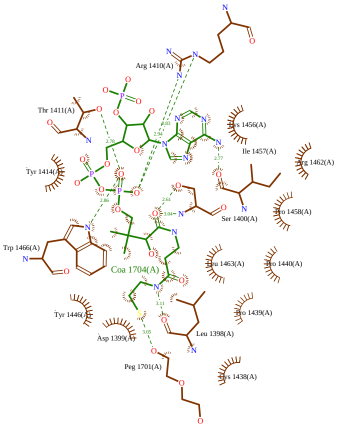

| protein | amino acids contacts (binding site) |
| --- | --- |
| PDB:4pzr:chainA:Q09472 | L D S R T Y C P P Y K I P R L W |
| sp:Q09472:EP300_HUMAN | L D S R T Y C P P Y K I P R L W |
| sp:Q92793:CBP_HUMAN | L D S R T Y C P P Y K I P R L W |
| tr:Q91XT0:Q91XT0_RAT | - - - - - - - - - - - - - - - - |
| sp:B2RWS6:EP300_MOUSE | L D S R T Y C P P Y K I P R L W |
| tr:O01368:O01368_DROME | L D S R T Y C P P Y K I P R L W |
| tr:Q9W321:Q9W321_DROME | L D S R T Y C P P Y K I P R L W |
| sp:P34545:CBP1_CAEEL | L D S R T Y C P P Y K I P R L W |
| sp:P35817:BDF1_YEAST | E D D D N I T S G S R K R A L E |

| | whole protein | | domain-based | | contact-based | |
| --- | --- | --- | --- | --- | --- | --- |
| protein | ident | simil | ident | simil | ident | simil |
| PDB:4pzr:chainA:Q09472 | 1.0 | 1.0 | 1.0 | 1.0 | 1.0 | 1.0 |
| sp:Q09472:EP300_HUMAN | 1.0 | 1.0 | 1.0 | 1.0 | 1.0 | 1.0 |
| sp:Q92793:CBP_HUMAN | 0.56 | 0.81 | 0.9 | 0.97 | 1.0 | 1.0 |
| tr:Q91XT0:Q91XT0_RAT | 0.14 | 0.14 | 0.0 | 0.0 | 0.0 | 0.0 |
| sp:B2RWS6:EP300_MOUSE | 0.94 | 0.98 | 0.99 | 1.0 | 1.0 | 1.0 |
| tr:O01368:O01368_DROME | 0.26 | 0.54 | 0.64 | 0.86 | 1.0 | 1.0 |
| tr:Q9W321:Q9W321_DROME | 0.26 | 0.56 | 0.64 | 0.86 | 1.0 | 1.0 |
| sp:P34545:CBP1_CAEEL | 0.27 | 0.56 | 0.57 | 0.85 | 1.0 | 1.0 |
| sp:P35817:BDF1_YEAST | 0.03 | 0.16 | 0.04 | 0.35 | 0.13 | 0.42 |

### nej (FBgn0261617) associated phenotypes

cell migration defective, chemical sensitive, developmental rate defective, dominant, gravitaxis defective, heat sensitive, hypoactive, lethal - all die before end of P-stage, lethal - all die before end of prepupal stage, lethal - all die before end of pupal stage, mitotic cell cycle defective, neuroanatomy defective, neurophysiology defective, partially lethal - majority die, partially lethal - majority live, somatic clone, some die during P-stage, some die during pharate adult stage, some die during prepupal stage, some die during pupal stage

(Information from FlyBase)

### cbp-1 (WBGene00000366) associated phenotypes

bag of worms, embryonic lethal, larval lethal, protruding vulva, sick, slow growth, sterile, vulvaless

(Information from WormBase)

### cbp-1 (UniProt:P34545) annotation

**Function**: Acetyltransferase enzyme. Acetylates histones, giving a specific tag for transcriptional activation (By similarity). May prevent DNA damage-induced apoptosis by inhibiting cep-1-dependent transcription activation of the programmed cell death activator egl-1 (PubMed:19521535). (UniProtKB:Q92793, PubMed:19521535).

**Subunit**: Interacts (via N-terminus domain and HAT domain) with prmt-5; the interaction results in methylation of cbp-1 (PubMed:19521535). Interacts (via HAT domain) with cep-1; cep-1 transcriptional activity may be inhibited by interaction with methylated cbp-1 (PubMed:19521535). Component of a complex that contains prmt-5 and cbp-1 (PubMed:19521535). (PubMed:19521535, PubMed:19521535).

**Subcellular location**: Nucleus (UniProtKB:Q92793).

**Ptm**: Methylation by prmt-5 may repress the capacity of cbp-1 to enhance cep-1-dependent transcription of egl-1. (PubMed:19521535).

(Information from UniProt)
